# Foster Kennedy Syndrome from Frontal Lobe Meningioma: A Rare Case Report

**DOI:** 10.22336/rjo.2025.43

**Published:** 2025

**Authors:** Rizaldy Taslim Pinzon, Marlyna Afifudin, Ananda Digdoyo, FX Kevin Christiansen Naibaho, Petra Gusti Parikesit, Yoel Sasamu Allendio

**Affiliations:** 1 Department of Neurology, Bethesda Hospital/ Faculty of Medicine, Duta Wacana Christian University, Yogyakarta, Indonesia; 2 Department of Ophthalmology, Bethesda Hospital/ Faculty of Medicine, Duta Wacana Christian University, Yogyakarta, Indonesia; 3 Faculty of Medicine, Duta Wacana Christian University, Yogyakarta, Indonesia

**Keywords:** meningioma, Foster Kennedy syndrome, frontal lobe, OS = Oculi Sinistra, OD = Oculi Dextra, ICP = Intracranial Pressure, RAPD = relative afferent pupillary defect, CBC = Complete Blood Count, CT = Computerized Tomography, MRI = Magnetic Resonance Imaging

## Abstract

**Background:**

Foster Kennedy syndrome is a neuro-ophthalmological disorder characterized by ipsilateral vision loss in one eye, followed by clinically significant papilledema in the opposite eye. The presence of mass lesions in the frontal lobe is primarily responsible for this syndrome. This case report further discusses symblepharon as an ocular manifestation of SJS.

**Method:**

A case report.

**Case report:**

We present a case of a 59-year-old female with a history of progressive headache, anosmia, mental status changes, and progressive poor vision. Ocular examination revealed disc pallor in her left eye with disc oedema in the contralateral eye. The patient was sent for computerized tomography (CT) and MRI, and the diagnosis of frontal lobe meningioma was confirmed. The surgical removal was performed, and the condition improved gradually.

**Discussion:**

We present a case of Foster Kennedy Syndrome, a rare neurological sign characterized by optic atrophy (vision loss) in one eye and papilledema (swelling of the optic nerve) in the other eye, often associated with an intracranial mass (meningioma).

**Conclusion:**

Presence of cranial fossa meningioma related to direct compression of a unilateral optic nerve, resulting in optic atrophy and might induce a rise in intracranial pressure, resulting in contralateral papilledema. This case presentation demonstrated that prompt and appropriate treatment was effective in gradually reducing the deterioration of symptoms associated with Foster Kennedy syndrome.

## Introduction

Foster Kennedy syndrome is a rare condition characterized by unilateral optic atrophy and contralateral disc oedema [1]. This rare syndrome is caused by direct compression of the ipsilateral optic nerve and olfactory nerve early, followed by secondarily increased intracranial pressure, which produces disc edema in the remaining intact optic nerve [1,2]. The most common causes are frontal lobe tumours and olfactory groove meningiomas [1-3]. Unilateral optic atrophy primarily results from an intracranial lesion exerting pressure on the optic nerve. The contralateral disc edema is a consequence of increased intracranial pressure [2,3].

The mass lesions that cause Foster Kennedy syndrome tend to occur in the frontal lobe, falx cerebri, olfactory groove, sphenoid wing, or subfrontal area [3]. We report a case of a 59-year-old female with a history of progressive headache, anosmia, mental status changes, and progressive poor vision. This case was confirmed as Foster Kennedy syndrome from frontal lobe meningioma.

## Case report

A 59-year-old right-handed woman underwent a neurology examination for a chronic progressive headache for approximately 4 months that had worsened over the 3 weeks before hospital admission. In addition, her left eye visual acuity (OS) had steadily deteriorated over the same period, resulting in almost total loss of monocular vision. She has shown no past episodes of nausea, vomiting, and seizures. She had no history of radiotherapy or recurrent head injuries. The family also complained of slowly progressive changes in mental status (forgetfulness and slow speech).

**Fig. 1:**
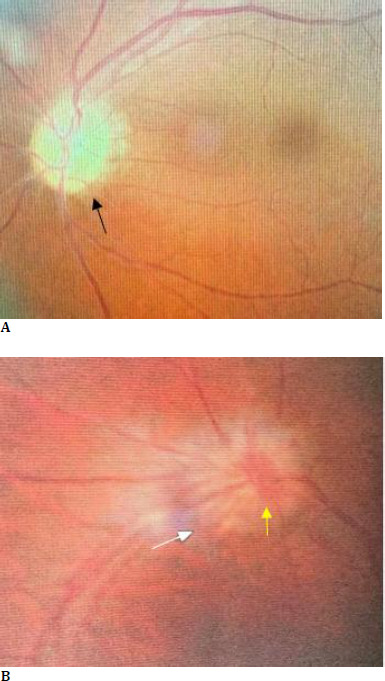
**A**. Fundoscopy showed pallor of the left optic disc (black arrow) or left-sided optic atrophy, and **B**. blurred margins of the right optic disc (white arrow) with disc hyperemia (yellow arrow) or right-sided papilledema

**Fig. 2:**
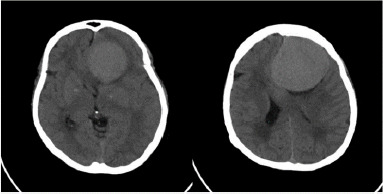
The brain CT showed a hyperdense mass lesion in the frontal lobe with significant oedema

**Fig. 3:**
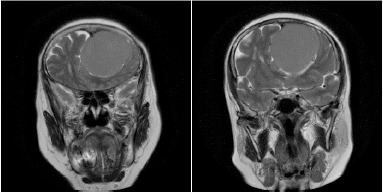
The brain MRI showed a frontal lobe isointense mass

**Fig. 4:**
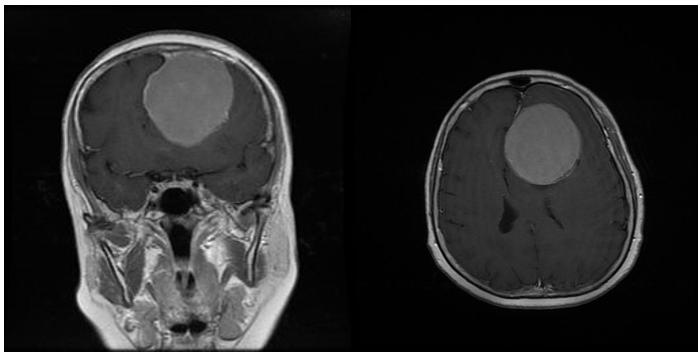
Contrast-enhanced mass in the frontal lobe consistent with meningioma

On her neurological examination, the patient had bilateral anosmia, no light perception in the left eye (OS), and 20/20 visual acuity in the right eye (OD). The patient also had a left relative afferent pupillary defect (RAPD). The ophthalmology consultation also revealed a pale left optic disc, indicative of papillary optic atrophy, and a blurred right optic disc border with disc hyperemia, also indicative of papillary changes (**Fig. 1**). The complete blood count (CBC) and metabolic profile were within normal limits. A brain computed tomography scan (CT scan) in the absence of contrast revealed a homogeneous mass measuring 4.7 cm × 4.2 cm × 3.4 cm in the left frontal lobe (**Fig. 2**). Further MRI on T1 sequence showed a large isointense mass with the left optic apex compressed followed by moderately oedematous, but with no evidence of hydrocephalus. Contract MRI showed enhancement consistent with meningioma (**Fig. 3,4**).

**Fig. 5:**
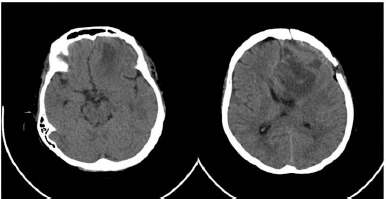
Complete removal of the tumour from serial brain CT

This patient was promptly sent for hospitalization and given a full dose of mannitol and steroids. Following an expert consultation at the Neurosurgery Department, emergency brain surgery was scheduled immediately. The complete removal of the mass confirmed the pathologic result of meningioma. The headache was completely resolved, and the blurred vision and mental status changes were also gradually improved. The serial CT scan showed complete removal of the brain tumour (**Fig. 5**).

## Discussion

We hereby present a case of Foster Kennedy syndrome, as previously documented by Kennedy in 1911. The syndrome is characterized by the clinical appearance of unilateral optic atrophy, accompanied by contralateral papilledema and anosmia. The most common mass lesions associated with Foster Kennedy syndrome are found in the falx cerebri, olfactory groove, sphenoid wings, and subfrontal areas [3,4]. Our case was associated with meningioma, and many publications state that meningioma remains the most frequent lesion [3-5].

Our case showed ipsilateral disc atrophy with contralateral disc edema. The ipsilateral optic atrophy is attributed to a process known as direct compartmental compression of a single optic nerve. This syndrome, in combination with elevated intracranial pressure (ICP), typically results in contralateral papilledema, which often arises before ipsilateral optic atrophy. The other common symptoms and signs were headaches, nausea and vomiting, emotional instability, loss of memory, or weakness. In our cases, progressive headache and forgetfulness were present.

Meningiomas constitute approximately 15% of all intracranial tumours and might appear as a single mass or several masses. Meningiomas can be caused by chromosomal loss, previous radiation exposure, or head trauma. During the reproductive years, the reported female-to-male ratio increases by a factor of 2:1. However, the potential association between the presence of hormonal receptors and the natural development of meningiomas has remained ambiguous [5]. In our case, we did not find any significant risk factors.

Brain CT scan and MRI have been identified as the preferred imaging modalities for the diagnosis of Foster Kennedy syndrome. In our case, the contrast MRI was consistent with meningioma. The compression directly on the disc was related to the presence of a cranial fossa meningioma, which could be located in the frontal lobe, olfactory groove, or sphenoid wing. It is noteworthy that substantial growth can exert pressure on the intracranial space, leading to contralateral papilledema. Advancing disease may result in ipsilateral atrophy of the optic nerve, characterized by a loss of nerve fibres and a reduction in symptoms of papilledema [6,7].

In suspected Foster Kennedy syndrome, the patient should be asked about their vision and whether it has decreased suddenly or gradually over time. The history should include probing to determine indications of elevated intracranial pressure, such as headache, diplopia, and nausea and vomiting. Anosmia and emotional disorientation are other symptoms that patients and families have reported. Furthermore, it is imperative to inquire about any behavioural changes in the patient’s family members [6-8]. In our patient, headaches, mental status changes, and blurred vision were present.

A fundus examination reveals the presence of ipsilateral optic atrophy and contralateral papilledema, providing diagnostic evidence of Foster-Kennedy syndrome [5-7]. In the majority of cases, the contralateral eye’s visual acuity is preserved until the disease progresses to an advanced or late stage. All patients suspected of having Foster Kennedy syndrome should undergo head and orbital CT scans, as well as MRIs with or without contrast, as neuroimaging findings are typically used to make the clinical diagnosis [5,6].

Foster Kennedy syndrome is usually treated with surgical resection. In terms of medical therapy, corticosteroids are commonly used as the first-line treatment to reduce intracranial pressure and edema surrounding the tumor [7,8]. In our cases, we gave a full dose of mannitol and steroid, followed by a complete surgical removal. The follow-up visit showed significant clinical improvement.

## Conclusion

We reported a rare case of Foster Kennedy syndrome (characterized by unilateral optic atrophy and contralateral disc oedema) from a large frontal lobe meningioma. The presence of a cranial fossa meningioma was associated with direct compression of a unilateral optic nerve and may induce a rise in intracranial pressure, resulting in contralateral papilledema. This case presentation demonstrated that prompt and appropriate treatment was effective in gradually reducing the deterioration of symptoms associated with Foster Kennedy syndrome.
